# Cross-Kingdom Small RNAs among Animals, Plants and Microbes

**DOI:** 10.3390/cells8040371

**Published:** 2019-04-23

**Authors:** Jun Zeng, Vijai Kumar Gupta, Yueming Jiang, Bao Yang, Liang Gong, Hong Zhu

**Affiliations:** 1 Key Laboratory of South China Agricultural Plant Molecular Analysis and Genetic Improvement, South China Botanical Garden, Chinese Academy of Sciences, Guangzhou 510650, China; junzeng@scbg.ac.cn; 2 University of Chinese Academy of Sciences, Beijing 100049, China; 3 Department of Chemistry and Biotechnology, School of Science, Tallinn University of Technology, 12618 Tallinn, Estonia; vijaifzd@gmail.com; 4 Key Laboratory of Plant Resource Conservation and Sustainable Utilization, Guangdong Provincial Key Laboratory of Applied Botany, South China Botanical Garden, Chinese Academy of Sciences, Guangzhou 510650, China; ymjiang@scbg.ac.cn (Y.J.); yangbao@scbg.ac.cn (B.Y.); 5 Key Laboratory of Post-Harvest Handling of fruits, Ministry of Agriculture, Guangzhou 510650, China

**Keywords:** RNA interference, cross-kingdom RNAi, small RNAs, crop protection, HIGS, SIGS

## Abstract

Small RNAs (sRNAs), a class of regulatory non-coding RNAs around 20~30-nt long, including small interfering RNAs (siRNAs) and microRNAs (miRNAs), are critical regulators of gene expression. Recently, accumulating evidence indicates that sRNAs can be transferred not only within cells and tissues of individual organisms, but also across different eukaryotic species, serving as a bond connecting the animal, plant, and microbial worlds. In this review, we summarize the results from recent studies on cross-kingdom sRNA communication. We not only review the horizontal transfer of sRNAs among animals, plants and microbes, but also discuss the mechanism of RNA interference (RNAi) signal transmission via cross-kingdom sRNAs. We also compare the advantages of host-induced gene silencing (HIGS) and spray-induced gene silencing (SIGS) technology and look forward to their applicable prospects in controlling fungal diseases.

## 1. Introduction

The sRNA-mediated RNA interference (RNAi) is a regulatory mechanism conserved in eukaryotes, where sRNAs play key roles in numerous biological processes, including RNA stability and processing, biotic and abiotic stress response and the regulation of morphological and developmental events [1,2,3]. The eukaryote transcriptomes are enriched in two types of endogenous small RNAs: small interfering RNAs (siRNAs) and microRNAs (miRNAs). siRNAs conventionally refer to a class of 20–24-nt double-stranded (dsRNA) molecules that are processed from longer precursors, deriving from the genome or exogenous RNA sequences such as viruses and transgene transcripts [4,5]. miRNAs are single-stranded non-coding RNAs of typically 20–22-nt in length produced from primary miRNAs (pri-miRNAs) containing a stem-loop structure, which is mostly transcribed from regions located between protein-coding genes [6,7]. In eukaryotes, both siRNA and miRNA are processed inside the cell by RNase III-like endonucleases named Drosha and/or Dicer, then bound by Argonaute (AGO) proteins and incorporated into RNA-induced silencing complex (RISC), which, in most cases, negatively regulates target gene expression at the post-transcriptional level [2,6,7]. In other cases, they can trigger the biogenesis of secondary siRNAs to amplify the silencing effect [8,9].

While RNAi has been discovered for more than 20 years, cross-kingdom sRNAs have only been reported quite recently [10,11,12,13,14]. In this report, we review the studies that describe the process and effect of siRNAs and miRNAs that move across animal, plant and microbe species. We also extensively discuss the mechanisms and factors for sRNA migration and silencing. Moreover, we present and evaluate the controversial view that plant-derived miRNAs can influence animal genes. Finally, we summarize and discuss the outlook for the application of RNAi technology for crop protection and human therapy.

## 2. Mobility of Small RNAs within an Organism

Effective migration of sRNAs is a basic premise for their silencing no matter whether they are specifically mobilized as an endogenous physiological response or overexpressed in organisms due to exogenous introduction. Once triggered within a single-cell type, the RNA-silencing signal can move from cell to cell and over long distances to alter gene expression in cells/tissues remote from the primary initiation sites, a phenomenon called ‘systemic acquired silencing’ (SAS) [15,16,17]. Cell-to-cell movement of sRNAs has been previously studied in plants, and it is likely that siRNA duplexes function as mobile silencing signals between plant cells [18]. Based on the studies of mammalian cells, there is a transfer of siRNAs and miRNAs between cells and tissues. Moreover, sRNA transfer appears to be a process of active selection for potentially functional sRNAs [19,20,21], since mobile sRNA profile is usually distinguished from the total sRNA population within the cells. Some factors such as AGOs, RNA-binding proteins (RBPs), wrapping into extracellular vesicles (EVs), or other transport machinery can also be involved in such sRNA selection process [22]. More recently, miRNA mobility is found to be precisely directed through a gating mechanism at specific cell-cell interface, restraining the long-distance shoot-to-root trafficking [23]. While some components and channels required for mobile silencing have been genetically deciphered in plants and nematodes, it remains an open question as to how possible and biologically significant the endogenous mobile silencing pathways are [15,17].

## 3. Horizontal Transfer of Mobile Small RNAs across Kingdoms

It has been recently noted that sRNA signals can be transmitted between different species (Table 1), revealing a new form of communication between distantly related organisms that interact, which is also called ‘cross-kingdom RNAi’ [24].

### 3.1. Cross-Kingdom sRNAs across Pathogens/Parasites and Host Animals

The sRNA traffic was firstly reported in 1998 when *Caenorhabditis elegans* were fed a dsRNA-expressing bacterial strain; siRNAs were ingested by the nematode and blocked its endogenous gene expression [25]. It has been described as ‘post-transcriptional gene silencing’ in plants and as ‘quelling’ in fungi [26,27]. Since the discovery of RNAi in animals, it has been widely used as a functional genomic technique to analyze gene function [28,29]. In addition to double-stranded siRNA, single-stranded miRNA has also been found to transfer between the host and the invasive species. For example, miRNAs deriving from parasites, such as *Schistosoma Japonicum* and *Litomosoides sigmodontis*, have been found in the body fluids of infected individuals [13,30]. Conversely, miRNA-mediated silencing signals can be transmitted in the opposite direction. Liu et al. identified the role of fecal miRNAs in shaping and manipulating gut microbiome in mice, where certain miRNAs could enter bacteria such as *Fusobacterium nucleatum* and *Escherichia coli*, specifically regulated bacterial gene transcripts and affected their growth. Loss-of-miRNA function mice exhibited uncontrolled gut microbiota and exacerbated colitis [31]. Similarly, it has been revealed that the resistance of sickle erythrocytes to malaria was partly enabled by miRNAs that could translocate into the parasite *Plasmodium falciparum* and interfere with its mRNA transcript, resulting in translational inhibition via impaired ribosomal loading [14].

### 3.2. Cross-Kingdom sRNAs from Pathogens/Parasites to Host Plants

**Figure 1:**
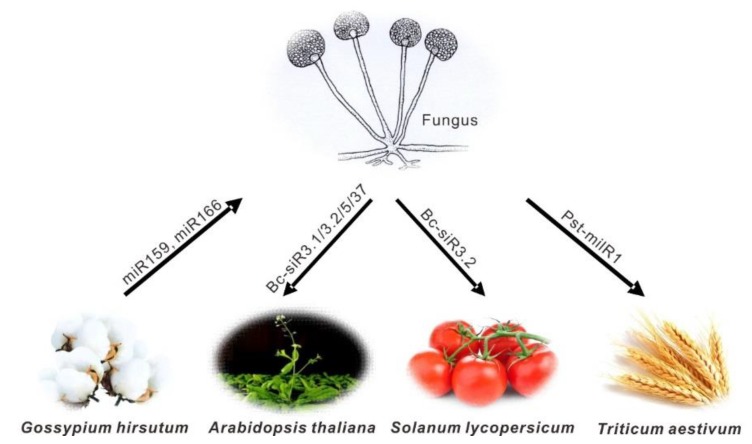
Cross-kingdom small regulatory RNAs in plant-pathogen interactions. Transfer of representative sRNAs between fungal pathogens and host plant species are presented. The arrows indicate the direction of the sRNA transfer.

Plant pathogenic fungi are the main factors that cause crop yield loss and affect global food security. Plants are susceptible to a broad spectrum of fungal pathogens. It has been reported that fungal sRNA molecules of *Botrytis cinerea* were transferred into host plant cells, acting as sRNA effectors to suppress host immunity and achieve infection [32]. This was the first time that fungal pathogen has been found to deliver sRNAs into host cells and hijack plant mRNAs, although it was known for decades that RNAs could carry long-distance signals in plants [33]. Upon the infection in *Arabidopsis*, *B. cinerea* Bc-siR3.2 could be loaded into AGO1 and targeted plant *mitogen-activated protein kinase 2* (*MPK2*) and *MPK1* transcripts, as well as the tomato *MPKKK4*, which are involved in plant immunity response to fungal pathogen attack [32]. Additionally, an oxidative stress-related gene, *peroxiredoxin* (*PRXIIF*) and *cell wall-associated kinase* (*WAK*) were targeted by Bc-siR3.1 and Bc-siR5, respectively [32]. Such sRNA effectors were mostly produced by Dicer-like protein 1 (Bc-DCL1) and Bc-DCL2 [34]. Moreover, Bc-siR37 targeted three immune responsive genes (*WRKY7*, *PMR6* and *FEI2*), which encode an immune-related transcription factor, a pectin lyase, and a leucine-rich repeat (LRR) receptor kinase, respectively. In a follow-up study, these target genes were suppressed in the transgenic *Arabidopsis* plants overexpressing Bc-siR37, which exhibited enhanced disease susceptibility to *B. cinerea* [35] (Figure 1).

Similar to the fungus-derived siRNAs, virus-derived dsRNAs can also be processed by Dicer-like (DCL) proteins into virus-derived siRNAs (vsiRNAs), which then guide AGO proteins to target host genes to mediate disease symptoms in plants [36,37]. Gene expression can be suppressed in a sequence-specific manner by infection with virus vectors carrying fragments from the exons of host plant genes [38]. The reverse genetic technology of virus-induced gene silencing (VIGS) has long been used in functional genomics studies by inhibiting the target gene expression. vsiRNAs originate predominantly from highly base-paired structures from the positive-strand viral genomic RNA [39]. In virus-infected plants, vsiRNAs can target transcripts at the post-transcriptional regulation level through sequence complementarity. It was reported that vsiRNAs from the Y-satellite of *Cucumber mosaic virus* specifically downregulated the mRNA of tobacco *ChII* gene, which induced a bright yellow mosaic symptom [40]. In addition, vsiRNAs derived from two grapevine-infecting viruses have been predicted to cleave host transcripts by deep sequencing. For example, vsiR1378 and vsiR6978 targeted transcripts encoding putative S2P metalloprotease and vacuolar protein-sorting55 (*VPS55*) [41]. In *Withania somnifera*, VIGS technology was established by silencing phytoene desaturase (*PDS*) and squalene synthase (*SQS*) in this slow-growing and difficult-to-transform plant. The silencing of *SQS* gene negatively regulated sterol and defense-related genes, leading to reduced phytosterols, withanolides and stress tolerance [42].

Intriguingly, a novel microRNA-like RNA 1 (Pst-milR1) from *Puccinia striiformis* f. sp. *tritici*, was exported into wheat and suppressed the wheat pathogenesis-related 2 (*PR2*) gene to impair wheat resistance to *Pst*. Silencing of the Pst-milR1 precursor resulted in increased wheat resistance to the virulent *Pst*, while *PR2* knockdown plants increased the susceptibility to *Pst* [43]. Consistent with Bc-siRNAs [32], Pst-milR1 may also function as an effector to inhibit the host plant immune defense response [43] (Figure 1). These studies suggest that both fungi and viruses can use cross-kingdom RNAi strategy to suppress the innate immune system of the host plants and ensure the success of their infection.

Compared to the sRNA transfer from pathogen to hosts, the functional movement of sRNAs from parasitic plant to host plants are rarely reported, until recently the parasitic plant *Cuscuta campestris* was found to deliver specific 22-nt miRNAs to suppress host messenger RNAs and trigger endogenous secondary siRNA production, and such miRNA delivery is likely to be applicable to a wide range of host plants, suggesting it as a universal strategy for plant parasitism [10].

### 3.3. Cross-Kingdom sRNAs from Host Plants to Pathogens/Parasites

Like fungi, viruses and parasitic plants that transfer sRNAs into host plants to suppress host immunity, host plants in nature can also export sRNAs into pathogenic invaders to induce gene silencing and reduce pathogenicity [44,45]. Comparatively, host-to-parasite movement of RNAi signals is less reported, likely due to the limited host range and insufficient efficacy of silencing [46]. More recently, the report on a naturally occurring miRNA trafficking between cotton plants and a fungal pathogen has further expanded the understanding of plant-delivered-sRNA silencing of pathogen virulence [12] (Figure 1). In this study, upon infection with *Verticillium dahliae*, cotton accumulated miR166 and miR159 that target *V. dahliae* genes encoding a Ca^2+^-dependent cysteine protease (*Clp-1*) and an isotrichodermin C-15 hydroxylase (*HiC-15*), respectively. These two miRNAs were exported both to fungal hyphae for specific silencing. More importantly, both *Clp-1* and *HiC-15* transcripts were reduced in the hyphae recovered from *V. dahliae*-infected cotton and the fungus mutants with targeted genes knocked out indeed displayed reduced virulence [12]. Meanwhile, it was noted that the sequences of *Clp-1* and *HiC-15* targeted respectively by miR166 and miR159 were highly conserved among different strains of *V. dahliae*, especially within the miRNA-binding regions [12]. These findings indicated that a fungal pathogen might have preserved or evolved this miRNA-dependent regulation to prevent host plant hypersensitive responses and to keep them alive during the biotrophic phase of the infection. It is also possible that pathogen transcripts are not targeted for cleavage by host miRNAs, in view of coevolution during a long-term specific host–pathogen interaction. This study also presented a description of a conserved host plant defense strategy against fungal pathogens by specifically downregulating virulence genes expression [12]. Another controversial case is that honeysuckle miR2911 could inhibit the synthesis of influenza A viruses (IAVs) through significantly decreasing target genes *PB2* and *NS1* expression level, which occurred in the human body. However, miR2911 is atypical since it exhibits unusual stability and does not follow the canonical mechanisms of miRNA biosynthesis and action [47,48].

### 3.4. Cross-Kingdom sRNAs across Plants and Animals (Insects/Mammals)

Plants have also been found to transfer double-stranded siRNAs to closely interacting insects to silence their transcripts and suppress their growth, also known as plant-mediated RNA interference (PM-RNAi) [49]. Commercially, the biotechnology solutions for controlling pests on crops depend on the expression of *Bacillus thuringiensis* (*Bt*) insecticidal proteins [50]. However, there is still urgent demand for highly potent targets to overcome the limitation of applicable insect species and the ever-increasing resistance. More recently, it has been revealed that *NDUFV2*, a subunit of mitochondrial complex I that catalyzes nicotinamide adenine dinucleotide (NADH) dehydrogenation in the respiratory chain, could serve as a promising target for precise insect control [51]. RNAi has been an alternative to control insect pests, but transferring sRNA into and between insect cells is still unraveled. However, two types of RNAi response have been recognized in different insect orders [52], one is systemic RNAi, which means the silencing effect is transported from the cell in which the dsRNA is applied or expressed to other cells, also to other tissues, in which the silencing will then take place, such as in the insects western corn rootworm [53] and Colorado Potato Beetle [54]. Another one is cell-autonomous, in which RNAi effects are limited to the cell which dsRNA is expressed or introduced, such as the insect *Drosophila melanogaster* [55]. In addition, the movement of plant miRNAs to animals has also been reported. Jia et al. conducted multiple assays and confirmed that mulberry-derived miRNAs could enter silkworm hemolymph and multiple tissues, although feeding silkworms with synthetic miR166b caused no phenotypic changes [56]. It is worth noting that plant miR162a could directly bind to the target gene *Apis mellifera TOR* (*amTOR*), which is essential for honeybee caste differentiation, thereby inhibiting larval ovary growth and inducing development into worker bees [57]. Therefore, horizontal cross-kingdom RNAi may open up an important area to further study the molecular mechanism of animal evolution.

While the transfer of sRNA silencing signals between plants and animals mainly through feeding has been reported, a highly debated issue remains as to whether there is a real transfer of dietary sRNAs from plants to mammals. In 2012, Zhang et al. first demonstrated the accumulation and biological function of dietary miRNAs in animal tissues [58]. Several follow-up studies generated either similar or contradictory results, and the focus of the debate is whether the dietary uptake of plant miRNAs into the mammal tissues is stable and biologically functional. The most tit-for-tat report came from Dickinson et al., who found that insignificant levels of rice miR168a did not result in a cross-kingdom modulation of low-density lipoprotein receptor adapter protein 1 (LDLRAP1) protein levels in mouse liver [59]. Similarly, plant miRNAs could hardly be detected in the plasma of healthy athletes and mice after ingestion of commonly consumed miRNA-rich food [60]. In addition, the apparent uptake of dietary plant miRNAs was not observed in the macaque blood by droplet digital PCR [61]. One major reason for these discrepancies seems to be the sRNA library construction and sequencing procedure. Plant miRNAs usually bear 2’-*O*-methylated 3’ ends [62], and this common modification has been reported to negatively influence the adaptor ligation efficiency, resulting in the underestimation of plant miRNAs compared with non-modified animal miRNAs [63]. Zhang’s team used oxidized deep sequencing to retrieve plant miRNAs in the serum of human and mice, while Dickinson et al. did not even detect enough plant miRNAs in the rice samples, indicating a bias for sequencing. Another argument is that plant-derived miRNAs could be contaminants during library preparation and sequencing, based on the fact that plant miRNAs are present in public animal sRNA database [64,65]. In experiments, it is common for nucleic acid cross contamination to cause false positive results. Therefore, whenever extremely low quantity of miRNAs is detected, the first consideration is if any contamination or background noise exits in the instrument. To reduce this possibility, multiple assay platforms, including deep sequencing, qRT-PCR and RNA gel blot, need to be applied for cross-validation.

Regardless of the contradiction, much experimental evidence has demonstrated the absorption and bioavailability of cross-species plant miRNAs [66,67,68]. More recently, it has been reported that plant-derived exosome-like nanoparticles (ELNs) containing sRNAs could alter microbiome composition and host physiology in mouse. Among these, ginger ELNs miR7267 could increase the yield of indole-3-carboxaldehyde in *Lactobacillus rhamnosus*, and these functions were linked to improve mouse colitis via IL-22-dependent manner [69]. Scientists also have started to specifically assess the function of dietary miRNAs for cancer therapy [70]. However, a recent study showed that transgenic miRNAs did not have any bioavailability, even though they were highly expressed and displayed digestive stability [71]. Taken together, the influence of plant-derived dietary miRNAs on the physiological progress of recipient animal organism remains to be carefully elucidated, in particular, the stoichiometry of the interactions between dietary miRNAs and their mammalian target genes should be taken into consideration [72,73,74]. Whether dietary miRNA can become a ‘rising star’ in cancer therapy is still open to question.

**Table 1:** Naturally occurring small RNAs and their target genes in cross-kingdom interactions.

sRNA	From	To	Target Genes	Reference
miR-515-5p	*H. sapiens/M. musculus*	*F. nucleatum*	16S rRNA	[31]
miR-1226-5p	*H. sapiens/M. musculus*	*E. coli*	*yegH*	[31]
Bc-siR3.2	*B. cinerea*	*A. thaliana*	*MPK2* and *MPK1*	[32]
Bc-siR3.1	*B. cinerea*	*A. thaliana*	*PRXIIF*	[32]
Bc-siR5	*B. cinerea*	*A. thaliana*	*WAK*	[32]
Bc-siR3.2	*B. cinerea*	*S. lycopersicum*	*MAPKKK4*	[32]
Bc-siR37	*B. cinerea*	*A. thaliana*	*WRKY7*, *PMR6* and *FEI2*	[35]
Pst-milR1	*P. striiformis* f. sp. *tritici*	*T. aestivum*	*PR2*	[43]
vsiR1378	GFkV	*V. vinifera*	S2P metalloprotease	[41]
vsiR6978	GRSPaV	*V. vinifera*	*VPS55*	[41]
miR166	*G. hirsutum*	*V. dahliae*	*Clp-1*	[12]
miR159	*G. hirsutum*	*V. dahliae*	*HiC-15*	[12]
miR2911	*L. japonica*	IAVs	*PB2* and *NS1*	[48]
miR162a	*B. campestris*	*A. mellifera*	*amTOR*	[57]
miR168a *	*O. sativa*	*H. sapiens/M. musculus*	LDLRAP1	[58]
miR159	*B. oleracea* var. *botrytis*	*H. sapiens*	*TCF7*	[70]

The ‘From’ and ‘To’ columns indicate the direction of RNAi transmission signals. * miR168a needs to be further validated in animal systems because of controversial studies [58,59]. The underlined *B. oleracea* var. *botrytis*, which is called broccoli, was particularly rich in miR159 by profiling the abundance of it in several commonly consumed plants and only a minority of miR159 was degraded after cooking, so we speculated that miR159 derived from broccoli.

## 4. Factors That Affect Cross-Kingdom sRNA Mechanism

With the discovery of cross-kingdom sRNAs, it becomes intriguing to explore how mobile sRNAs move across the boundary of different kingdoms. One point for the mechanistic aspect of cross-kingdom RNAi is to assess the dose effect of the transferred sRNAs. In worms or pathogens, an amplification pathway exists to allow a small number of initial sRNAs to generate abundant secondary sRNAs and trigger an extensive response. In mammals, however, the sRNAs need to be absorbed in sufficient amounts to achieve significant effects [75]. On the other hand, exogenous sRNAs usually encounter harsh biological environment, including RNases, phagocytosis and extreme-pH, etc. Therefore, to guarantee the efficacy of transmitted sRNAs, their stability really matters. Extracellular vesicles (EVs) are essential vehicles of intercellular communication and they largely perform the function of protecting sRNAs [76,77]. It has been speculated that some sRNA molecules may travel to the fungi via an exosomal pathway since exosomes accumulate at plant-fungus contact sites and vesicles fusion is observed [78]. Additionally, plant multivesicular bodies have been shown to contain small RNAs and other necessary components of the silencing machinery [79]. A recent study in *Arabidopsis* revealed that plant cells could secrete exosome like EVs to deliver sRNAs into *B. cinerea* [80]. During the evolutionary arms race with fungal pathogens, *Arabidopsis* has evolved EVs-mediated cross-kingdom RNAi as a unique method to active its immune responses [80]. Moreover, miRNAs from commercial dairy cow milk were found to be resistant to digestion and associated mostly with EVs, which appeared to be potentially bioavailable [81]. Some miRNAs in bovine-milk exosomes may regulate the expression of human genes [82]. EV sRNA cargos are delivered to affect the local immune response and manipulate target cell gene expression. Substantial evidence points to EVs as universal carriers of extracellular RNA from bacteria, archaea, fungi, and protists [83]. However, whether EV pathway conserves in all forms of life requires further elucidation.

In addition to the EV encapsulation, other recognition and/or modification machinery may play important roles in preventing mobile sRNAs from degradation during the transfer process [24,77]. For instance, sRNAs in the mammalian blood are found to form a circulating complex with AGO2, which are not just membrane-enveloped as previously proposed [84,85]. Similarly, RNA-binding protein (RBP)-associated trafficking system may contribute to the stability of serum miRNAs in the mammalian extracellular environment, making them as potential biomarkers for disease diagnosis [86,87]. Moreover, similar to the cellular RNAs that usually undergo modifications for increased diversity and functional potential after transcription, plant miRNAs have been characterized to be methylated on the 3’ terminal nucleotide after miRNA/miRNA* duplex formation. Additionally, this modification is believed to protect miRNAs from 3’ terminal uridylation that might trigger their degradation in vivo [62,88]. However, viruses from fungi and oomycetes had evolved a counter defense strategy against RNA silencing in their hosts, which was called an RNA silencing suppressor (RSS), such as in the white root rot fungus, *Rosellinia necatrix* [89] and in the oomycete *Phytophthora infestans* [90].

## 5. HIGS and SIGS

**Figure 2:**
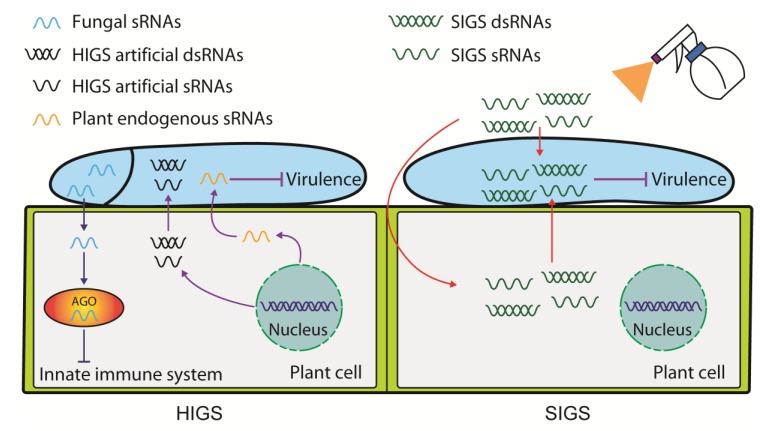
Host-induced gene silencing (HIGS) and spray-induced gene silencing (SIGS) for controlling fungal pathogens. This schematic diagram illustrates the transmission of cross-kingdom RNAi signals in plant-fungal pathogen interactions, and how HIGS and SIGS can be used to protect plants against fungal infection. On the left panel, fungal pathogens deliver sRNA effectors into host plant cells and hijack the host innate immune system (blue arrows and blue block sign). To react, the host plant cells also export either endogenous sRNAs or artificial sRNAs into pathogen cells to silence virulence genes and other important genes for fungi growth (purple arrows and purple block sign). On the right panel, SIGS sRNAs or long dsRNAs, which target fungal pathogenicity-related genes, can be either taken up directly by pathogen cells, or indirectly move from hosts that uptake them to pathogen cells (red arrows).

Exogenously introduced sRNAs via overexpression in organisms due to engineering modified plant, virus or laboratory introduction would be highly relevant for RNA-based agricultural applications if they exert expected functions. Host-induced gene silencing (HIGS), based on the RNAi principle, has been widely used as an important disease-control method, where specific virulence factors of the pathogens and/or viruses can be silenced by host-derived sRNAs designed to target these genes [78,91]. As a highly efficient genetic strategy for controlling sucking insects, nematodes and pathogenic fungi [92], HIGS technology does not require the cultivation of disease-resistant plants. This technology was first applied by Huang et al. to silence a root-knot nematode parasitism gene by expressing dsRNA in *Arabidopsis* [93]. Indeed, it has long been considered as an effective tool to address fungi gene function and control fungal diseases [78,94,95,96]. For example, Xu et al. used *Tobacco rattle virus* (TRV)-based RNAi constructs in cotton plants to silence a regulator of G-protein signaling gene of invaded *V. dahliae* and enhance resistance to this pathogen [94]. Similarly, Song et al. assessed whether three *V. dahliae* virulence genes (*Ave1*, *Sge1* and *NLP1*) could be used to inhibit *Verticillium* wilt as silencing targets by transiently expressing *TRV*::*RNAi* constructs in tomato. Subsequently, only HIGS of *Sge1* was not achieved because of a light reduction in *Sge1* expression [97]. Recently, a report on the naturally transferred miRNAs from cotton to *V. dahliae* for silencing has further expanded its application [12]. In all, depending on the suitable target gene chosen, HIGS against pathogens is operational and can be applied to plant protection, especially for the crop plants that are ecologically important with high agronomic values. It is also reasonable to believe that HIGS has the potential to accurately control multiple diseases by using transgenic plants that express multiple stacked RNAi target sequences, excluding off-targets in the given crop (Figure 2).

Since *B. cinerea* has been shown to take up external sRNAs and dsRNAs, an alternative technology called spray-induced gene silencing (SIGS), has also been effective for crop protection against pathogens by spraying sRNAs and dsRNAs that target fungal genes on the surface of fruits, vegetables and flowers [34,98]. A recent study demonstrated for the first time that spraying barley long dsRNAs that target *Fusarium graminearum* cytochrome P450 lanosterol C-14α-demethylase (*CYP51*) gene significantly inhibited the fungal growth. The exogenous long dsRNA was taken up by the plant and transferred in an unmodified form via the vascular system to fungal infection sites where it was processed into siRNAs by fungal DCL1 for its antifungal activity [99]. However, much remains unknown about how exogenous RNAs are taken up by plant and fungal cells, and how these RNAs are transferred from plant cells into fungal cells [100]. Fortunately, Song et al. (2018) revealed that fungal dsRNAs in plant cells could efficiently turn into substantial siRNAs via plant RNAi machinery, and then deliver into fungal cells to induce RNAi machinery in *Fusarium* spp. fungi [101]. We summarized the successful application cases of SIGS-mediated gene silencing for the control of plant pathogens (Table 2). More extensive attempts on insect RNAi have been reviewed elsewhere [102]. Compared with HIGS, the knowledge of SIGS is still limited, and more exploration is needed. Overall, there are still obstacles for using SIGS, due to sRNA degradation, cell wall barrier and host plant diversity, etc., which hamper the development of new broad-spectrum environment-friendly fungicides into mass production. Therefore, there is still a long way to go from laboratory to practical applications. Encouragingly, the nano-biotechnology has recently improved the potential applications of SIGS for crop protection. A recent study showed that non-toxic, degradable, layered double hydroxide (LDH) clay nanosheets could carry large amounts of dsRNAs for sustained and effective protection against plant viruses for at least 20 days after application [103]. The LDH delivery system that maintains a stable and constant release of dsRNA provides an excellent tool to gain fundamental insights into the mechanism of dsRNA systemic transport. Different from spraying LDH nanosheets on the leaf surface, another study reported that DNA nanostructures could internalize into plant cells through cell walls and deliver siRNAs to mature plant tissues without external mechanical aid, and effectively silence gene expression in tobacco leaves [104]. This study determines the feasibility of DNA nanostructures that deliver biomolecules to plant cells. In summary, nanotechnology can serve as a promising tool set for siRNA delivery to plants for efficient gene silencing, as has proven valuable in human therapies [105,106].

## 6. Concluding Remarks

While it is common for organisms in biological niches to exchange RNA-silencing signals, many mechanistic aspects of these signals need further investigation to understand better how a given biological equilibrium is obtained during the sRNA crosstalk. Revelations may come from the two main aspects, i.e., sRNA generation and secretion from the producing cells, and sRNA recognition and uptake by the recipient cells. Cross-kingdom sRNAs hold a big promise for pest and disease control, but it is still part of the process to find lethal genes suitable for the RNAi-based technologies in microbial pathogens or pests, as well as effective delivery strategies for sRNA direct application in the natural environments. Inspiringly, the first drug based on RNAi therapy has been approved by U.S. Food and Drug Administration (FDA) in 2018, which is expected to push the treatment of human diseases to a new level [108]. With the rapid development of deep sRNA sequencing technology and comparative genomics, more cross-kingdom sRNAs will be discovered, along with the effectors. There are also many mysteries concerning sRNA-mediated cross-kingdom gene regulation. For instance, how are endogenous and exogenous sRNAs distinguished by an individual RNAi system and then function in multiple signaling pathways? Can animal sRNAs affect plant growth and development in cross-kingdom RNAi? What roles do sRNAs play in coevolution? Answering these questions would reveal the detailed mechanism of bidirectional RNAi signal transmission, to facilitate a full understanding of sRNAs in nature.

## Figures and Tables

**Table 2:** SIGS-mediated gene knockdown in plant-pathogen interactions.

Target Pathogen	Host Plant	Concentration of dsRNA	Target Gene	Reference
*B. cinerea*	*A. thaliana*, etc.	20 ng/μL	*DCL1/2*	[34]
*V. dahliae*	*A. thaliana*	20 ng/μL	*DCL*	[34]
*F. graminearum*	*H. vulgare*	20 ng/μL	*CYP51*	[99]
*F. asiaticum*	*T. aestivum*	0.1 pM	*Myosin 5*	[101]
*S. sclerotiorum*	*B. napus*	20 ng/μL *	SS1G_01703, etc.	[107]
*S. sclerotiorum*	*A. thaliana*	20 ng/μL	SS1G_03208, etc.	[107]
*B. cinerea*	*B. napus*	42 ng/μL	BC1G_04955, etc.	[107]

* In this study, senescing petals of *B. napus* were first incubated with 20 ng/μL dsRNA for three days, and then another dsRNA solution (8 ng/μL) was applied to the leaf surface of plants at approximately 30–50% flowering stage.

## Abbreviations

AGO	argonaute
DCL	dicer-like
dsRNA	double-stranded RNA
ELNs	exosome-like nanoparticles
EVs	extracellular vesicles
HIGS	host-induced gene silencing
LDH	layered double hydroxide
miRNAs	microRNAs
PM-RNAi	plant-mediated RNA interference
RBPs	RNA-binding proteins
RISC	RNA-induced silencing complex
RNAi	RNA interference
SIGS	spray-induced gene silencing
siRNAs	small interfering RNAs
sRNA	small RNA
VIGS	virus-induced gene silencing
